# Out-of-Pocket Spending for Insulin, Diabetes-Related Supplies, and Other Health Care Services Among Privately Insured US Patients With Type 1 Diabetes

**DOI:** 10.1001/jamainternmed.2020.1308

**Published:** 2020-06-01

**Authors:** Kao-Ping Chua, Joyce M. Lee, Rena M. Conti

**Affiliations:** 1 Susan B. Meister Child Health Evaluation and Research Center, Department of Pediatrics, University of Michigan Medical School, Ann Arbor; 2 Division of Pediatric Endocrinology, Department of Pediatrics, University of Michigan Medical School, Ann Arbor; 3 Institute for Health System Innovation and Policy, Questrom School of Business, Department of Markets, Public Policy, and Law, Boston University, Boston, Massachusetts

## Abstract

This study evaluates inpatient, outpatient, and pharmacy claims to identify the annual out-of-pocket expenditures for both insured children and adults with type 1 diabetes.

Concern about high out-of-pocket spending for insulin has prompted states, payers, and pharmacy benefit managers to limit cost-sharing in the US.^1,2^ In addition to insulin, patients with type 1 diabetes might have out-of-pocket expenses for other care, such as diabetes-related supplies. Using national 2018 claims data, we estimated out-of-pocket spending for insulin and other health care services for privately insured patients with type 1 diabetes.

## Methods

We analyzed 2018 data from the IBM MarketScan Commercial Database,^3^ which included patients with employer-sponsored coverage from medium to large firms. We included patients aged 1 to 64 years with continuous enrollment throughout 2018, 1 or more type 1 diabetes diagnosis code in 2017 (to limit the analysis to established patients), and 1 or more insulin claim in 2018. Because the data used were deidentified, the institutional review board of the University of Michigan Medical School did not regulate this study as human participant research; informed consent was not required.

We assigned inpatient, outpatient, and pharmacy claims in 2018 to 4 categories: (1) insulin, (2) diabetes-related supplies (eg, insulin pumps, glucometers), (3) other type 1 diabetes–related services (medications such as sulfonylureas and inpatient/outpatient claims with a type 1 diabetes diagnosis code), and (4) all other services not assigned to the first 3 categories (eg, visits for viral illness) (eAppendixes 1-4 in the Supplement). Based on whether patients had 1 or more claim for insulin pumps or associated supplies, and whether patients had 1 or more claim for continuous glucose monitors or associated supplies, we classified patients as using pumps only, continuous glucose monitors only, both, or neither.

For each category, we calculated the mean (SD) and median annual out-of-pocket spending. We calculated the proportion of annual overall out-of-pocket spending accounted for by insulin. We also calculated out-of-pocket spending for diabetes-related supplies according to use of insulin pumps and continuous glucose monitors.

In subgroup analyses, we stratified by age (children aged 1 to 17 years vs adults aged 18 years or older) and enrollment in a high-deductible health plan, defined as qualified high-deductible health plans with statutorily defined minimum deductibles of US $1350 for individual and US $2700 for family coverage in 2018 or consumer-driven health plans that typically couple high deductibles with health reimbursement arrangement accounts.^4^

## Results

The 65 192 patients who met the inclusion criteria had a mean (SD) age of 40.8 (16.5) years and included 7842 children (12.0%), 30 133 women (46.2%), and 14 680 individuals (22.5%) enrolled in high-deductible health plans. Overall, 37 042 patients (56.8%) used insulin pumps, continuous glucose monitors, or both.

The use of diabetes technologies and annual out-of-pocket spending are shown in the Table. Mean (SD) annual out-of-pocket spending for insulin was lower ($435 [$544]) than spending for diabetes-related supplies ($490 [$785]). Mean (SD) annual overall out-of-pocket spending was $2414 ($3531); for 5191 patients (8.0%), this spending exceeded $5000. Insulin accounted for 18.0% of overall out-of-pocket spending. Mean (SD) annual out-of-pocket spending for diabetes-related supplies varied among patients who used insulin pumps only ($562 [$626]), continuous glucose monitors only ($472 [$625]), both ($1037 [$1039]), or neither ($79 [$175]).

**Table.** Diabetes Technology Use and Annual Out-of-Pocket Spending Among Privately Insured Patients With Type 1 Diabetes^a^

Outcome	All patients (n = 65 192)	Children (n = 7842)	Adults (n = 57 350)	Patients enrolled in high-deductible health plans^b^	
				Yes (n = 14 680)	No (n = 50 512)
**Technology use, No. (%)**					
Insulin pumps but not continuous glucose monitors	8626 (13.2)	1075 (13.7)	7551 (13.2)	1947 (13.3)	6679 (13.2)
Continuous glucose monitors but not insulin pumps	8083 (12.4)	1391 (17.7)	6692 (11.7)	2052 (14.0)	6031 (11.9)
Both insulin pumps and continuous glucose monitors	20 333 (31.2)	3698 (47.2)	16 635 (29.0)	4866 (33.2)	15 467 (30.6)
Patients using neither insulin pumps nor continuous glucose monitors	28 150 (43.2)	1678 (21.4)	26 472 (46.2)	5815 (39.6)	22 335 (44.2)
**Out-of-pocket spending, US$**					
Insulin					
Mean (SD)	435 (544)	497 (664)	427 (524)	568 (667)	397 (495)
Median (25th-75th percentile)	280 (100-560)	315 (120-640)	278 (100-554)	375 (80-804)	270 (105-501)
Diabetes-related supplies^c^					
Mean (SD)	490 (785)	823 (977)	445 (744)	688 (988)	433 (705)
Median (25th-75th percentile)	102 (0-678)	446 (23-1306)	80 (0-588)	188 (0-1097)	88 (0-589)
Other type 1 diabetes–related services					
Mean (SD)	385 (788)	510 (824)	368 (781)	542 (783)	340 (783)
Median (25th-75th percentile)	188 (67-427)	295 (153-592)	174 (60-402)	300 (94-645)	168 (61-368)
All other services^d^					
Mean (SD)	1103 (3248)	468 (991)	1190 (3434)	1335 (1721)	1036 (3569)
Median (25th-75th percentile)	537 (164-1424)	192 (52-535)	613 (196-1560)	698 (183-1899)	502 (160-1297)
Overall					
Mean (SD)	2414 (3531)	2298 (1817)	2430 (3704)	3132 (1916)	2205 (3851)
Median (25th-75th percentile)	1993 (1055-3301)	1977 (1038-3224)	1995 (1056-3314)	2904 (1770-4119)	1764 (935-2958)
Overall out-of-pocket spending accounted for by insulin, %	18.00	21.60	17.60	18.10	18.00

^a^ Data from IBM Watson Health.^3^

^b^ Based on whether the MarketScan plan type variable indicated enrollment in a consumer-driven health plan or qualified high-deductible health plan throughout 2018.

^c^ Includes insulin pumps/supplies (eg, pump unit, infusion sets), continuous glucose monitors/supplies (eg, sensors, transmitters, receivers), glucometers/supplies (eg, meter, lancets, glucose strips), urine testing strips, blood ketone tests, needles, and syringes.

^d^ Inpatient, outpatient, or pharmacy claims that were not for insulin, diabetes-related supplies, or other type 1 diabetes–related services.

Compared with adults, children had higher use of diabetes technologies and higher out-of-pocket spending for diabetes-related supplies (Table). Among children, out-of-pocket spending for diabetes-related supplies ($823) was higher than for insulin ($497) (Figure). Among adults, out-of-pocket spending for diabetes-related supplies ($445) and insulin ($427) were similar. Patients enrolled in high-deductible health plans had higher out-of-pocket spending in each category than patients enrolled in other plans (Table).

**Figure.**
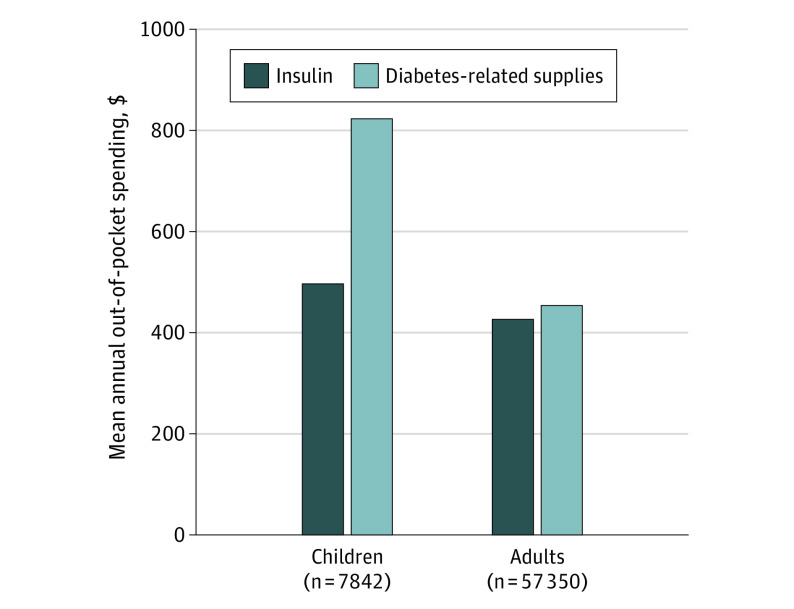
Mean Annual Out-of-Pocket Spending for Insulin and Diabetes-Related Supplies Among Privately Insured Children and Adults With Type 1 Diabetes Diabetes-related supplies include insulin pumps and supplies (eg, pump unit, infusion sets), continuous glucose monitors and supplies (eg, sensors, transmitters, and receivers), glucometers and supplies (eg, meter, lancets, glucose strips), urine testing strips, blood ketone tests, needles, and syringes. Data from IBM Watson Health.^3^

## Discussion

In a national sample of privately insured patients with type 1 diabetes, mean out-of-pocket spending for all care was nearly $2500 in 2018. For 8% of patients, this spending exceeded $5000. Insulin accounted for 18% of all out-of-pocket spending and less out-of-pocket spending than diabetes-related supplies. Findings suggest that substantial out-of-pocket burden may remain for patients with type 1 diabetes even if insulin cost-sharing is limited. Government officials and insurers should consider improving coverage for all type 1 diabetes–related services, following the approach of a 2019 rule allowing qualified high-deductible health plans to cover services such as insulin and glucometers before deductibles are met.^5^

This study has limitations. We did not study patients covered by Medicare or Medicaid or those without insurance. The database included patients who received insurance from medium to large firms,^3^ which typically offer plans with lower deductibles compared with plans offered by small firms.^6^ Consequently, findings may underestimate out-of-pocket spending for patients with type 1 diabetes who receive insurance from small firms.

## Notes

Supplement. **eAppendix 1.** Methodology for Identifying Insulin Claims **eAppendix 2.** CPT/HCPCS Codes for Diabetes-Related Supplies **eAppendix 3.** National Drug Codes for Diabetes-Related Supplies **eAppendix 4.** Anti-Diabetes Medications
