# Supplementary material for: Out-of-Pocket Spending for Insulin, Diabetes-Related Supplies, and Other Health Care Services Among Privately Insured US Patients With Type 1 Diabetes
Source: JAMA Intern Med. 2020 Jun 1;180(7):1012–4. doi: 10.1001/jamainternmed.2020.1308 (PMC7265118; doi:10.1001/jamainternmed.2020.1308)
Supplement: Supplement. — eAppendix 1. Methodology for Identifying Insulin Claims eAppendix 2. CPT/HCPCS Codes for Diabetes-Related Supplies eAppendix 3. National Drug Codes for Diabetes-Related Supplies eAppendix 4. Anti-Diabetes Medications [file jamainternmed-180-1012-s001.pdf]

Supplementary Online Content

Chua KP, Lee JM, Conti RM. Out-of-pocket spending for insulin, diabetes-related supplies, and other health care services among privately insured US patients with type 1 diabetes. *JAMA Intern Med.* Published online June 1, 2020. doi:10.1001/jamainternmed.2020.1308

eAppendix 1. Methodology for Identifying Insulin Claims

eAppendix 2. CPT/HCPCS Codes for Diabetes-Related Supplies

eAppendix 3. National Drug Codes for Diabetes-Related Supplies

eAppendix 4. Anti-Diabetes Medications

This supplementary material has been provided by the authors to give readers additional information about their work.

© 2020 Chua KP et al. *JAMA Internal Medicine.*

eAppendix 1. Methodology for Identifying Insulin Claims

Using the 2018 IBM MarketScan Red Book, we compiled national drug codes for drugs that had the string "insulin" in the generic drug name and that had a therapeutic class of 172 (Antidiabetic Agents, Insulin) or 238 (Pharmaceutical Aids/Adjuv, NEC). We also included national drug codes for OmniPod Pods and OmniPod Dash 5 Pack Pods (pre-filled insulin pods for use in this pump system). Product names are listed below.

ADMELOG
AFREZZA
APIDRA
APIDRA SOLOSTAR
BASAGLAR KWIKPEN
EXUBERA
EXUBERA COMBINATION PACK 12
EXUBERA COMBINATION PACK 15
EXUBERA KIT
FIASP
FIASP FLEXTOUCH
HUMALOG
HUMALOG JUNIOR KWIKPEN
HUMALOG KWIKPEN
HUMALOG MIX 50/50
HUMALOG MIX 50/50 KWIKPEN
HUMALOG MIX 75/25
HUMALOG MIX 75/25 KWIKPEN
HUMALOG MIX 75/25 PEN
HUMALOG PEN
HUMULIN
HUMULIN 50/50
HUMULIN 70/30
HUMULIN 70/30 KWIKPEN
HUMULIN 70/30 PEN
HUMULIN BR
HUMULIN L
HUMULIN N (NPH)
HUMULIN N (NPH) KWIKPEN
HUMULIN N PEN
HUMULIN R
HUMULIN R CONCENTRATED U-500
HUMULIN R CONCENTRATED U-500 KWIKPEN
HUMULIN U
ILETIN II LENTE BEEF
ILETIN II LENTE PORK
ILETIN II NPH BEEF

ILETIN II NPH PORK
ILETIN II PZI BEEF
ILETIN II PZI PORK
ILETIN II REG. BEEF
ILETIN II REG. PORK
ILETIN II REGULAR PORK
ILETIN LENTE I
ILETIN NPH I
ILETIN PORK NPH
ILETIN PZI
ILETIN REGULAR I
ILETIN SEMILENTE
ILETIN ULTRALENTE
INSULATARD HUMAN INSULIN
INSULIN BEEF
INSULIN BOVINE
INSULIN HUMAN REGULAR
INSULIN LISPRO
INSULIN LISPRO KWIKPEN
INSULIN PURIFIED
INSULIN PURIFIED LENTE PORK
INSULIN PURIFIED NPH PORK
INSULIN PURIFIED REGULAR PORK
INSULIN STANDARD LENTE
INSULIN STANDARD NPH
INSULIN STANDARD REGULAR
INSULIN STANDARD SEMILENTE
INSULIN STANDARD ULTRALENTE
INSULIN, PURIFIED SEMILENTE PORK
INSULIN, PURIFIED ULTRALENTE BEEF
LANTUS
LANTUS NOVAPLUS
LANTUS SOLOSTAR
LANTUS SOLOSTAR PEN
LANTUS SOLOSTAR PEN NOVAPLUS
LEVEMIR

LEVEMIR FLEXPEN
LEVEMIR FLEXTOUCH
LISPRO-PFC
MIXTARD HUMAN INSULIN 70/30
MYXREDLIN
NOVOLIN 70/30
NOVOLIN 70/30 FLEXPEN
NOVOLIN 70/30 INNOLET
NOVOLIN 70/30 PENFILL
NOVOLIN L
NOVOLIN N
NOVOLIN N (NPH)
NOVOLIN N INNOLET
NOVOLIN N PENFILL
NOVOLIN R
NOVOLIN R INNOLET
NOVOLIN R PENFILL
NOVOLOG
NOVOLOG FLEXPEN
NOVOLOG MIX 70/30
NOVOLOG MIX 70/30 FLEXPEN
NOVOLOG PENFILL
OMNIPOD PODS
OMNIPOD DASH 5 PACK PODS
RELION HUMULIN 70/30
RELION HUMULIN N
RELION HUMULIN R
RELION NOVOLIN 70/30
RELION NOVOLIN 70/30 FLEXPEN
RELION NOVOLIN 70/30 INNOLET
RELION NOVOLIN N INNOLET
RELION NOVOLIN R
SOLIQUA 100/33
TOUJEO
TRESIBA
VELOSULIN BR
XULTOPHY 100/3.6

© 2020 Chua KP et al. *JAMA Internal Medicine.*

eAppendix 2. CPT/HCPCS Codes for Diabetes-Related Supplies

An endocrinologist on the study team (JL) and the primary author (KC) jointly classified whether the following CPT/HCPCS codes were related to continuous glucose monitors, insulin pumps, neither, or both (for artificial pancreas systems). We used the HCPCS codes below as well as national drug codes for diabetes-related supplies covered under the pharmacy benefit **(Appendix 3)** to assign patients to the four categories of diabetes technology utilization. Patients with a claim for an artificial pancreas system were assigned to the "insulin pump therapy/continuous glucose monitoring" category.

**HCPCS Code**	**Description**	**CGM, Pump, Neither, or Both**
A9276	Sensor; invasive (e.g., subcutaneous), disposable, for use with interstitial continuous glucose monitoring system, 1 unit = 1 day supply	CGM
A9277	Transmitter; external, for use with interstitial continuous glucose monitoring system	CGM
A9278	Receiver (monitor); external, for use with interstitial continuous glucose monitoring system	CGM
K0553	Supply allowance for therapeutic continuous glucose monitor (cgm), includes all supplies and accessories, 1 month supply = 1 unit of service	CGM
K0554	Receiver (monitor); external, for use with therapeutic glucose continuous monitor system	CGM
S1030	Continuous noninvasive glucose monitoring device, purchase (for physician interpretation of data, use CPT code)	CGM
S1031	Continuous noninvasive glucose monitoring device, rental, including sensor, sensor replacement, and download to monitor (for physician interpretation of data, use CPT code)	CGM
S1034	Artificial pancreas device system (e.g., low glucose suspend [LGS] feature) including continuous glucose monitor, blood glucose device, insulin pump and computer algorithm that communicates with all of the devices	Both
A4222	Infusion supplies for external drug infusion pump, per cassette or bag (list drugs separately)	Pump
A4224	Supplies for maintenance of insulin infusion catheter, per week	Pump
A4225	Supplies for external insulin infusion pump, syringe type cartridge, sterile, each	Pump
A4230	Infusion set for external insulin pump, nonneedle cannula type	Pump
A4231	Infusion set for external insulin pump, needle type	Pump
A4232	Syringe with needle for external insulin pump, sterile, 3 cc	Pump
A9274	External ambulatory insulin delivery system, disposable, each, includes all supplies and accessories	Pump
E0784	External ambulatory infusion pump, insulin	Pump
J1817	Insulin for administration through DME (i.e., insulin pump) per 50 units	Pump
K0601	Replacement battery for external infusion pump owned by patient, silver oxide, 1.5 volt, each	Pump
K0602	Replacement battery for external infusion pump owned by patient, silver oxide, 3 volt, each	Pump
K0603	Replacement battery for external infusion pump owned by patient, alkaline, 1.5 volt, each	Pump
K0604	Replacement battery for external infusion pump owned by patient, lithium, 3.6 volt, each	Pump
K0605	Replacement battery for external infusion pump owned by patient, lithium, 4.5 volt, each	Pump
S9353	Home infusion therapy, continuous insulin infusion therapy; administrative services, professional pharmacy services, care coordination, and all necessary supplies and equipment (drugs and nursing visits coded separately), per diem	Pump
82948	Glucose; blood, reagent strip	Neither
82962	Glucose, blood by glucose monitoring device(s) cleared by the FDA specifically for home use	Neither
A4233	Replacement battery, alkaline (other than J cell), for use with medically necessary home blood glucose monitor owned by patient, each	Neither
A4234	Replacement battery, alkaline, J cell, for use with medically necessary home blood glucose monitor owned by patient, each	Neither

© 2020 Chua KP et al. *JAMA Internal Medicine.*

A4235	Replacement battery, lithium, for use with medically necessary home blood glucose monitor owned by patient, each	Neither
A4236	Replacement battery, silver oxide, for use with medically necessary home blood glucose monitor owned by patient, each	Neither
A4250	Urine test or reagent strips or tablets (100 tablets or strips)	Neither
A4252	Blood ketone test or reagent strip, each	Neither
A4253	Blood glucose test or reagent strips for home blood glucose monitor, per 50 strips	Neither
A4255	Platforms for home blood glucose monitor, 50 per box	Neither
A4256	Normal, low, and high calibrator solution/chips	Neither
A4258	Spring-powered device for lancet, each	Neither
A4259	Lancets, per box of 100	Neither
A4772	Blood glucose test strips, for dialysis, per 50	Neither
A9275	Home glucose disposable monitor, includes test strips	Neither
E0607	Home blood glucose monitor	Neither
E2100	Blood glucose monitor with integrated voice synthesizer	Neither
E2101	Blood glucose monitor with integrated lancing/blood sample	Neither
A4206	Syringe with needle, sterile, 1 cc or less, each	Neither
A4207	Syringe with needle, sterile 2 cc, each	Neither
A4208	Syringe with needle, sterile 3 cc, each	Neither
A4209	Syringe with needle, sterile 5 cc or greater, each	Neither
A4213	Syringe, sterile, 20 cc or greater, each	Neither
A4215	Needle, sterile, any size, each	Neither
A4657	Syringe, with or without needle, each	Neither
S8490	Insulin syringes (100 syringes, any size)	Neither
S5565	Insulin cartridge for use in insulin delivery device other than pump; 150 units	Neither
S5566	Insulin cartridge for use in insulin delivery device other than pump; 300 units	Neither

© 2020 Chua KP et al. *JAMA Internal Medicine.*

eAppendix 3. National Drug Codes for Diabetes-Related Supplies

We included all national drug codes in the 2018 IBM MarketScan Red Book for:

- Products in MarketScan therapeutic class 85 (diabetes supplies)
- Products that had the string “insulin” in the generic name and that was in therapeutic class 237 (devices) or 999 (other)
- Products that had “Acetone test” or “Urine test, multiple” in the generic drug name
- Products that had “Infusion pump, insulin”, “Infusion pump, parenteral”, “Pump”, or “Pump, infusion” in the generic name.

We de-duplicated the resulting list of national drug codes and deleted products with the following brand names:

ARGYLE CLINICAL PRODUCTS EMERSON PUMP ADAPTER
CADD-LEGACY PORTABLE INFUSION PUMP
COMPANION CLEARSTAR PUMP
CONTROLLER LIFECARE
LIFECARE PUMP MODEL 4 PIGGYBACK
MEGAPUMP
MICRO PUMP LIFECARE
PLUM LC 5000
POWER KIT DISPOSABLE
PRECISION AIRLESS PUMP
SPORTGUARD PROTECTIVE CASE
OMNIPOD PODS
OMNIPOD DASH 5 PACK PODS
HEALTHCHECK URINARY TRACT INFECTION
HOMECHEK REVEAL MULTI-DRUG TESTING CUP
LEUKOSTIX
MICROSTIX-NITRITE
PHENISTIX
TEK-CHEK
URI-TEST NITRITE HOME SCREENING KIT
UTI HOMESCREENING TEST KIT
UTI HOMESCREENING TEST STICK
CARETOUCH BLOOD PRESSURE MONITOR

We also deleted products with the following generic names:
DEXTROSE
INACTIVE RECORD

For the resulting list of 4,548 national drug codes, an endocrinologist on the study team (JL) and the primary author (KC) jointly classified whether the product was related to continuous glucose monitors, insulin pumps, neither, or both, similar to our approach for CPT/HCPCS codes **(Appendix 2).** Below, we have included the 49 national drug codes for products related to continuous glucose monitors and the 93 codes for products related to insulin pumps (none were classified as being related to both). The full list of 4,406 national drug codes for supplies unrelated to continuous glucose monitors or insulin pumps is too long to include in this Appendix, but is available upon request to the corresponding author. These supplies included blood ketone tests, glucometers and associated supplies (e.g., glucometer, control solution, lancets, glucose testing strips), urine testing strips, and pens/needles/syringes.

© 2020 Chua KP et al. *JAMA Internal Medicine.*

Because Red Book is cumulative, the list of national drug codes includes some products that have been discontinued. No claims will be observed for such codes.

NDC number	Product Name	CGM, Pump, Neither, or Both
08627001031	DEXCOM G4 PLATINUM RECEIVER	CGM
08627002011	DEXCOM G4 PLATINUM RECEIVER	CGM
08627002021	DEXCOM G4 PLATINUM RECEIVER	CGM
08627002031	DEXCOM G4 PLATINUM RECEIVER	CGM
08627003011	DEXCOM G4 PLATINUM PEDIATRIC RECEIVER	CGM
08627003021	DEXCOM G4 PLATINUM PEDIATRIC RECEIVER	CGM
08627003031	DEXCOM G4 PLATINUM PEDIATRIC RECEIVER	CGM
08627005011	DEXCOM G4 PLATINUM RECEIVER	CGM
08627005021	DEXCOM G4 PLATINUM RECEIVER	CGM
08627005031	DEXCOM G4 PLATINUM RECEIVER	CGM
08627006011	DEXCOM G4 PLATINUM PEDIATRIC RECEIVER	CGM
08627006021	DEXCOM G4 PLATINUM PEDIATRIC RECEIVER	CGM
08627006031	DEXCOM G4 PLATINUM PEDIATRIC RECEIVER	CGM
08627008011	DEXCOM G5 RECEIVER	CGM
08627008021	DEXCOM G5 RECEIVER	CGM
08627008031	DEXCOM G5 RECEIVER	CGM
08627009011	DEXCOM RECEIVER KIT	CGM
08627009111	DEXCOM G6 RECEIVER	CGM
57599000021	FREESTYLE LIBRE 10-D READER	CGM
57599000200	FREESTYLE LIBRE 14 DAY READER	CGM
76300000211	SOF-SENSOR	CGM
76300000214	SOF-SENSOR	CGM
76300070021	SENSOR	CGM
76300750001	SEN-SERTER	CGM
76300000805	ENLITE GLUCOSE SENSOR	CGM
76300751001	ENLITE GLUCOSE SENSOR	CGM
08197100110	AUTOSENSOR	CGM
08197100116	AUTOSENSOR	CGM
08197200101	GLUCOWATCH G2 BIOGRAPHER	CGM
08627005104	DEXCOM G5/G4 PLATINUM SENSOR	CGM
08627005114	DEXCOM G4 PLATINUM SENSOR	CGM
08627005303	DEXCOM G6 SENSOR	CGM
08627074104	SEVEN SYSTEM SENSOR PACK	CGM
57599000019	FREESTYLE LIBRE 10-D SENSOR	CGM
57599000101	FREESTYLE LIBRE 14 DAY SENSOR	CGM
76300000204	SOF-SENSOR GLUCOSE SENSOR	CGM
08627073001	SEVEN PLUS CONTINUOUS GLUCOSE MONITORING SYSTEM	CGM
08627073101	SEVEN PLUS CONTINUOUS GLUCOSE MONITORING SYSTEM	CGM
08627073201	SEVEN PLUS CONTINUOUS GLUCOSE MONITORING SYSTEM	CGM
76300007201	GUARDIAN REAL-TIME SYSTEM	CGM
76300007202	GUARDIAN REAL-TIME SYSTEM PEDIATRIC	CGM

© 2020 Chua KP et al. *JAMA Internal Medicine.*

08627000301	DEXCOM G4 PLATINUM	CGM
08627001011	DEXCOM G4 PLATINUM	CGM
08627001021	DEXCOM G4 PLATINUM	CGM
08627001101	DEXCOM G4 PLATINUM	CGM
08627004104	DEXCOM G4 PLATINUM	CGM
08627001301	DEXCOM G4 PLATINUM TRANSMITTER	CGM
08627001401	DEXCOM G5 TRANSMITTER	CGM
08627001601	DEXCOM G6 TRANSMITTER	CGM
76300038610	QUICK-SET PARADIGM	Pump
76300038710	QUICK-SET PARADIGM	Pump
76300039410	QUICK-SET PARADIGM	Pump
76300084010	PARADIGM SURE-T INFUSION SET	Pump
76300012201	PARADIGM INSULIN PUMP PATHWAY PROGRAM	Pump
76300022201	PARADIGM INSULIN PUMP PATHWAY PROGRAM	Pump
76300052201	PARADIGM INSULIN PUMP PATHWAY PROGRAM	Pump
76300037710	SILHOUETTE PARADIGM INFUSION SET	Pump
76300038110	SILHOUETTE PARADIGM INFUSION SET	Pump
76300038210	SILHOUETTE PARADIGM INFUSION SET	Pump
08173106201	D-TRON ADAPTER	Pump
65781031010	ANIMAS PUMP CARTRIDGE	Pump
76300010310	MINIMED SYRINGE RESERVOIR	Pump
76300010324	MINIMED SYRINGE RESERVOIR	Pump
76300031221	PARADIGM POLYFIN QR WITH WINGS	Pump
76300031222	PARADIGM POLYFIN QR WITH WINGS	Pump
76300032012	SOF-SET MICRO NON-NEEDLE INFUSION	Pump
76300032112	SOF-SET MICRO NON-NEEDLE INFUSION	Pump
76300032610	PARADIGM RESERVOIR	Pump
76300033210	PARADIGM RESERVOIR	Pump
76300103100	MINIMED SYRINGE RESERVOIR	Pump
76300326100	PARADIGM RESERVOIR	Pump
76300332100	PARADIGM RESERVOIR	Pump
76300092110	MIO INFUSION SET	Pump
76300092310	MIO INFUSION SET	Pump
76300092510	MIO INFUSION SET	Pump
76300094110	MIO INFUSION SET	Pump
76300094310	MIO INFUSION SET	Pump
76300094510	MIO INFUSION SET	Pump
76300096510	MIO INFUSION SET	Pump
76300097510	MIO INFUSION SET	Pump
08508200000	OMNIPOD DASH SYSTEM	Pump
76300051511	PARADIGM 515	Pump
76300051512	PARADIGM 515	Pump
76300051513	PARADIGM 515	Pump
76300051514	PARADIGM 515	Pump

© 2020 Chua KP et al. *JAMA Internal Medicine.*

76300052211	PARADIGM 522	Pump
76300052212	PARADIGM 522	Pump
76300052213	PARADIGM 522	Pump
76300052214	PARADIGM 522	Pump
76300052219	PARADIGM 522	Pump
76300052220	PARADIGM 522	Pump
76300071211	PARADIGM 712	Pump
76300071212	PARADIGM 712	Pump
76300071213	PARADIGM 712	Pump
76300071214	PARADIGM 712	Pump
76300071511	PARADIGM 715	Pump
76300071512	PARADIGM 715	Pump
76300071513	PARADIGM 715	Pump
76300071514	PARADIGM 715	Pump
76300072211	PARADIGM 722	Pump
76300072212	PARADIGM 722	Pump
76300072213	PARADIGM 722	Pump
76300072214	PARADIGM 722	Pump
76300072219	PARADIGM 722	Pump
76300072220	PARADIGM 722	Pump
76300515110	PARADIGM 515	Pump
76300515120	PARADIGM 515	Pump
76300515130	PARADIGM 515	Pump
76300515140	PARADIGM 515	Pump
76300712110	PARADIGM 712	Pump
76300712120	PARADIGM 712	Pump
76300712130	PARADIGM 712	Pump
76300712140	PARADIGM 712	Pump
76300715110	PARADIGM 715	Pump
76300715120	PARADIGM 715	Pump
76300715130	PARADIGM 715	Pump
76300715140	PARADIGM 715	Pump
85081014002	OMNIPOD STARTER KIT	Pump
65781011001	ANIMAS INSULIN PUMP R1000	Pump
65781011101	ANIMAS INSULIN PUMP R1000	Pump
65781011201	ANIMAS INSULIN PUMP R1000	Pump
76300040713	MINIMED INFUSION PUMP	Pump
76300050812	MINIMED INSULIN INFUSION PUMP	Pump
76300050813	MINIMED INSULIN INFUSION PUMP	Pump
76300050818	MINIMED INSULIN INFUSION PUMP	Pump
76300050821	MINIMED INSULIN INFUSION PUMP	Pump
76300055111	MINIMED 530G	Pump
76300055112	MINIMED 530G	Pump
76300055113	MINIMED 530G	Pump

© 2020 Chua KP et al. *JAMA Internal Medicine.*

76300055114	MINIMED 530G	Pump
76300055115	MINIMED 530G	Pump
76300075111	MINIMED 530G	Pump
76300075112	MINIMED 530G	Pump
76300075113	MINIMED 530G	Pump
76300075114	MINIMED 530G	Pump
76300075115	MINIMED 530G	Pump
76300051111	PARADIGM INSULIN INFUSION PUMP	Pump
76300051112	PARADIGM INSULIN INFUSION PUMP	Pump
76300051113	PARADIGM INSULIN INFUSION PUMP	Pump
76300511110	PARADIGM INSULIN INFUSION PUMP	Pump
76300511120	PARADIGM INSULIN INFUSION PUMP	Pump
76300511130	PARADIGM INSULIN INFUSION PUMP	Pump

© 2020 Chua KP et al. *JAMA Internal Medicine.*

eAppendix 4. Anti-Diabetes Medications

Using the 2018 IBM MarketScan Red Book, we identified all products in MarketScan therapeutic class 173 (173-Antidiabetic Agents, Sulfonylureas), 174 (Antidiabetic Agents, Miscellaneous), 266 (Antidiabetic Agent, Meglitinides), 267 (Antidiabetic Agents, SGLT Inhibitors), and 268 (Antidiabetic Agent, TZD). Medications are listed below.

**Sulfonylureas**
ACETOHEXAMIDE
CHLORPROPAMIDE
GLIMEPIRIDE
GLIMEPIRIDE/PIOGLITAZONE HYDROCHLORIDE
GLIMEPIRIDE/ROSIGLITAZONE MALEATE
GLIPIZIDE
GLYBURIDE
GLYBURIDE, MICRONIZED
GLYBURIDE/METFORMIN HYDROCHLORIDE
TOLAZAMIDE
TOLBUTAMIDE
**Miscellaneous anti-diabetic agents**
ACARBOSE
ALBIGLUTIDE
ALOGLIPTIN BENZOATE
ALOGLIPTIN BENZOATE/METFORMIN HYDROCHLORIDE
ALOGLIPTIN BENZOATE/PIOGLITAZONE HYDROCHLORIDE
CANAGLIFLOZIN/METFORMIN HYDROCHLORIDE
DAPAGLIFLOZIN PROPANEDIOL/METFORMIN HYDROCHLORIDE
DAPAGLIFLOZIN/SAXAGLIPTIN
DULAGLUTIDE
EMPAGLIFLOZIN/METFORMIN HYDROCHLORIDE
EXENATIDE
GLIPIZIDE/METFORMIN HYDROCHLORIDE
GLUCAGON
GLUCAGON HYDROCHLORIDE
LINAGLIPTIN
LINAGLIPTIN/METFORMIN HYDROCHLORIDE
LIRAGLUTIDE
LIXISENATIDE
LIXISENATIDE;LIXISENATIDE
METFORMIN HCL;MEDICAL FOOD
METFORMIN HYDROCHLORIDE
METFORMIN HYDROCHLORIDE/PIOGLITAZONE HYDROCHLORIDE
METFORMIN HYDROCHLORIDE/REPAGLINIDE
METFORMIN HYDROCHLORIDE/ROSIGLITAZONE MALEATE
METFORMIN HYDROCHLORIDE/SAXAGLIPTIN HYDROCHLORIDE

© 2020 Chua KP et al. *JAMA Internal Medicine*.

METFORMIN HYDROCHLORIDE/SITAGLIPTIN PHOSPHATE
MIGLITOL
PRAMLINTIDE ACETATE
SAXAGLIPTIN HYDROCHLORIDE
SEMAGLUTIDE
SITAGLIPTIN PHOSPHATE
**SGLT inhibitors**
CANAGLIFLOZIN
DAPAGLIFLOZIN PROPANEDIOL
EMPAGLIFLOZIN
EMPAGLIFLOZIN/LINAGLIPTIN
ERTUGLIFLOZIN
ERTUGLIFLOZIN/METFORMIN HYDROCHLORIDE
ERTUGLIFLOZIN/SITAGLIPTIN
**Meglitinides**
NATEGLINIDE
REPAGLINIDE
**Thiazolidinediones**
PIOGLITAZONE HYDROCHLORIDE
ROSIGLITAZONE MALEATE
TROGLITAZONE

© 2020 Chua KP et al. *JAMA Internal Medicine.*
